# Beyond boundaries: Navigating the positive potential of ChatGPT, empowering education in underdeveloped corners of the world

**DOI:** 10.1016/j.heliyon.2024.e35845

**Published:** 2024-08-08

**Authors:** Amna Shabbir, Safdar Rizvi, Muhammad Mansoor Alam, Mazliham Mohd Su'ud

**Affiliations:** a Department of Electronic Engineering, NED University of Engineering & Technology, Karachi, Pakistan; b Department of Computer Science, Bahria University, Karachi Campus, Pakistan; c Faculty of Computer and Information, Multimedia University, Cyberjaya, Malaysia; d Riphah International University, Islamabad, Pakistan

**Keywords:** ChatGPT, Artificial intelligence, Higher education, Authentic student work, Ethical writing

## Abstract

In November 2022, ChatGPT3, an advanced AI-powered chatbot, emerged suddenly, generating significant interest in higher education. Concerns arose regarding its potential to complicate the authentication of genuine student work. While some foresaw negative impacts, we advocate a positive outlook, suggesting educators can utilize ChatGPT to cultivate supportive learning environments that enhance students' character development. Our study thoroughly explores ChatGPT's implications for education in underdeveloped countries, examining both opportunities and challenges within AI research in education. We investigate practical applications, and potential benefits, and propose responsible integration strategies for students, teachers, and schools in utilizing ChatGPT for learning and assessment. Emphasizing ethical use, we stress leadership, character development, and authentic assessment as crucial factors. Despite concerns about academic integrity, we highlight ChatGPT's dual nature, it can facilitate cheating but also has the potential to deepen learning experiences. Our research focuses on understanding ChatGPT's impact on education from both student and teacher perspectives, discussing future trends in learning and teaching. The strategic integration of ChatGPT and AI in universities demands ethical foresight, personalized learning strategies, and ongoing research to optimize educational benefits while preserving core values and fostering student development.

## 1 Introduction

Artificial Intelligence (AI) continues to evolve rapidly, with the Generative Pretrained Transformer (GPT) model, introduced by OpenAI in 2018, marking a significant advancement in Large Language Models (LLMs) [1]. This paper centers on the latest iterations of this model, specifically, ChatGPT-3.5 and ChatGPT-4, released in late 2022 and early 2023, respectively [2]. ChatGPT integrates deep learning and advanced algorithms to emulate human-like language processing abilities, excelling in text generation, question answering, and translation [3,4].

**Fig. 1:**
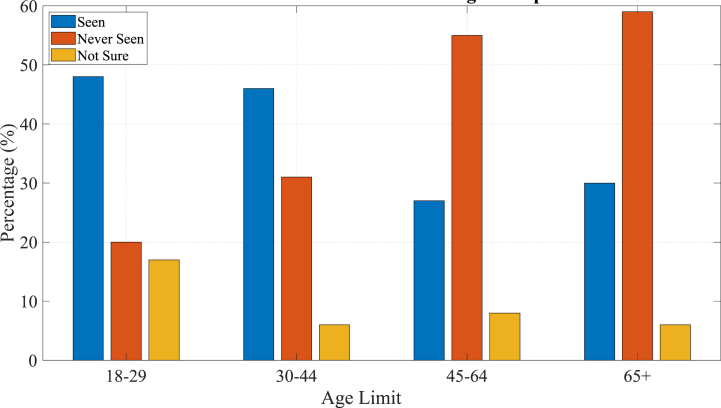
ChatGPT Awareness across age groups.

Moreover, recent data highlight as shown in Fig. 1, varying levels of familiarity with ChatGPT among university students across different age groups. Among 18-29-year-olds, 48 % have seen text generated by ChatGPT for others, underscoring a high level of exposure among undergraduate and some master's students. This familiarity suggests a readiness to integrate such technologies into academic activities, potentially enhancing personalized learning and administrative efficiency. In contrast, for 30-44-year-olds, where 46 % have observed ChatGPT-generated text and 31 % report no exposure, efforts are needed to introduce this cohort, primarily consisting of master's and PhD students, to the benefits of ChatGPT in research and specialized academic tasks.[1].

Amidst the growing adoption of ChatGPT and similar AI-driven technologies like Bing Chat, Bard, and Ernie in education [2, 3] [5]) It is crucial to comprehensively analyze its implications. Gurrib [4] surveyed existing machine learning (ML) applications in the finance industry and found benefits in terms of human resources and efficiency in financial decision-making, with, however, some key challenges in data quality, including understanding and interpretation of ML models. Kamalov et al. [6] provide an extensive review of the potential impact of AI on education, focusing on artificial intelligence applications in collaborative teacher-student learning, intelligent tutoring systems, automated assessment, and personalized learning. Drawing on insights from academics and practitioners, this study explores the transformative potential of ChatGPT in higher education. This paper adopts a theoretical lens, specifically the constructivist learning theory, to elucidate how ChatGPT can enhance active, collaborative, and personalized learning experiences in higher education. This theoretical framework underscores the pedagogical implications of integrating ChatGPT into educational practices. The structure of this paper unfolds as follows.

1. Challenges of ChatGPT for Higher Education: Examining critical challenges associated with ChatGPT's integration in higher education, supported by propositions.
2. Proposed Framework: Introducing a framework based on identified propositions to guide future research and practice in utilizing ChatGPT in higher education.

This paper navigates through the multifaceted impact of ChatGPT on higher education, delving into potential benefits, addressing challenges, and proposing a framework for informed decision-making at the intersection of artificial intelligence and academia.

## 2 Literature review

The literature review on the integration of ChatGPT in educational settings is crucial for understanding both the opportunities and challenges, particularly in the context of underdeveloped countries. In these regions, where access to traditional learning materials is often constrained, Yan's [7] concern about students excessively relying on ChatGPT gains heightened significance. Plagiarism concerns intersect with limited access to educational resources, potentially exacerbating issues of academic misconduct. The responsible use advocated by Anders [8] becomes imperative, emphasizing the need for students to treat ChatGPT as an assistive tool rather than a shortcut, especially in environments where traditional learning resources are scarce.

The potential hindrance to long-term cognitive development and creativity, as highlighted by Rudolph et al. [5], takes on unique dimensions in underdeveloped countries. Here, limitations in educational infrastructure and resources may compound the challenges faced by students who overly depend on ChatGPT, impeding their ability to independently accumulate knowledge. Moreover, the shift away from seeking assistance from traditional sources, as noted by Arif et al. [9], may be amplified in regions where educational support systems are under-resourced. The diminished development of collaboration and communication skills, critical for success in various professional fields, becomes a pressing concern in the context of underdeveloped countries.

In higher education within these regions, the worries expressed by academics about the potential misuse of AI, including ChatGPT, may intersect with resource constraints ([10]; 2021). Implementing technology to monitor and address academic misconduct linked to AI may pose additional challenges in environments where institutions already grapple with limited resources. The financial and temporal costs associated with such implementations could strain already stretched academic resources.

Despite these challenges, the potential benefits of ChatGPT in enhancing student engagement, satisfaction, and learning outcomes ([11]; Winkler & Söllner, 2018) become particularly relevant in underdeveloped countries. In settings where access to high-quality educational resources is limited, ChatGPT could potentially serve as a valuable tool to bridge gaps in learning opportunities. However, scholars' cautions about misinformation generation, bias, and privacy concerns (Malinka et al., 2023) need to be considered in the specific socioeconomic and cultural contexts of these regions.

The rapid proliferation of ChatGPT in education, with the emergence of GPT4, prompts a critical examination of its impact on learning in underdeveloped countries. Research in this area could provide insights into how these technologies can be effectively leveraged to overcome educational challenges while acknowledging and addressing the unique socioeconomic and infrastructural constraints faced by these nations.

**Fig. 2:**
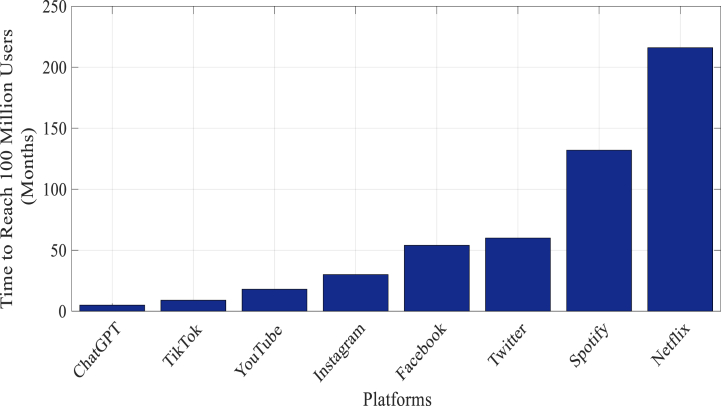
100M user milestone platform adoption rates [4].

From Fig. 2, when looking at how different platforms grew and got 100 million users. Netflix, a popular streaming service, took 10 years to reach this number because people were slowly getting used to watching on-demand videos in the late '90s. Social media platforms like Facebook, Twitter, WhatsApp, and Instagram, on the other hand, grew faster, reaching 100 million users in 3.5 to 2.5 years. Then there's Spotify, a music streaming service that started in 2008. It took Spotify a longer time, 11 years, to get 100 million users. The data also shows how quickly new platforms can become popular. TikTok, a short video app, reached 100 million users in just 0.75 years because people liked its short videos.

Now, let's talk about ChatGPT. It's the latest one on the list and uses advanced technology. In academia, which means in schools and research, ChatGPT became popular fast, getting 100 million users in only 0.1667 years. This shows that more and more people in academics are using ChatGPT for things like writing help and research assistance. So, the figure not only tells us how different platforms grew but also shows us that advanced AI tools like ChatGPT are becoming important in education and research.

Table 1, provides a comparative analysis for various platforms to reach 100 million users, showcasing distinctive patterns in user adoption. Notably, established streaming giant Netflix required a decade to reach this milestone, indicative of the gradual uptake in the late '90s for on-demand video services. In contrast, social media platforms demonstrated swifter growth, with Facebook, Twitter, WhatsApp, and Instagram achieving 100 million users in 3.5 to 2.5 years. Music streaming service Spotify, launched in 2008, followed a more extended trajectory, taking 11 years to reach the same user count. The data also highlights the rapid ascent of newer entrants. TikTok, a short-form video platform, attained 100 million users in a remarkably brief 0.75 years (9 months), reflecting the explosive popularity of its content format. Furthermore, with the focus on the rapid rise of ChatGPT, a cutting-edge AI technology, achieved the significant milestone of 100 million users in just 0.1667 years (2 months), showcasing its swift adoption in educational and research settings. This suggests that within a short period, ChatGPT has become widely embraced in academia, where its advanced capabilities contribute to tasks such as research, writing assistance, and other knowledge-driven activities.

**Table 1:** Advantages and disadvantages of ChatGPT in education.

Reference	Benefits	Drawbacks
Yan [7]	Provides opportunities for access to learning materials in regions with limited traditional resources.	Heightened concern about excessive reliance leading to potential academic misconduct and plagiarism.
Anders (2023)	Advocates responsible use, emphasizing ChatGPT as an assistive tool in resource-constrained environments.	Risk of students treating ChatGPT as a shortcut rather than a supplemental learning tool.
Rudolph et al. [5]	Potential benefits in overcoming educational challenges.	Potential hindrance to long-term cognitive development and creativity, especially if students overly depend on ChatGPT.
Arif et al. [9]	Acknowledges the potential for shifting educational support systems.	Amplification of the shift away from traditional sources may lead to diminished collaboration and communication skills.
Allen et al. [10]; 2021)	Recognition of potential benefits but expresses concerns about the misuse of AI, considering resource constraints.	Additional challenges in monitoring and addressing academic misconduct linked to AI in resource-limited environments.
Deng & Yu [11]; Winkler & Söllner (2018)	Potential for enhancing student engagement, satisfaction, and learning outcomes.	Need to address cautions about misinformation, bias, and privacy concerns in specific socioeconomic and cultural contexts.
Malinka et al. (2023)	Acknowledges potential benefits but highlights concern about misinformation, bias, and privacy in underdeveloped regions.	Need for careful consideration and mitigation of identified concerns in the unique contexts of underdeveloped countries.
Kasneci et al. (2023); Sullivan et al. (2023)	Calls for a nuanced perspective, considering both benefits and challenges.	Integration requires a careful balancing act to navigate the specific socioeconomic and infrastructural constraints.

In conclusion, the integration of ChatGPT in education in underdeveloped countries requires a nuanced perspective that considers both the potential benefits and the specific challenges associated with these contexts, as highlighted by the existing literature (Kasneci et al., 2023; Sullivan et al., 2023). Table 1, Summarizes the key benefits and drawbacks of integrating ChatGPT in education.

## 3 Research questions

This research initiative is designed to delve into the primary benefits and challenges encountered by students, teachers, and university administrators in underdeveloped countries as they integrate ChatGPT into their academic activities. Additionally, the study seeks to evaluate potential strategies for managing the use of ChatGPT in academic settings, considering the unique educational landscape and challenges prevalent in underdeveloped nations.

**Fig. 3:**
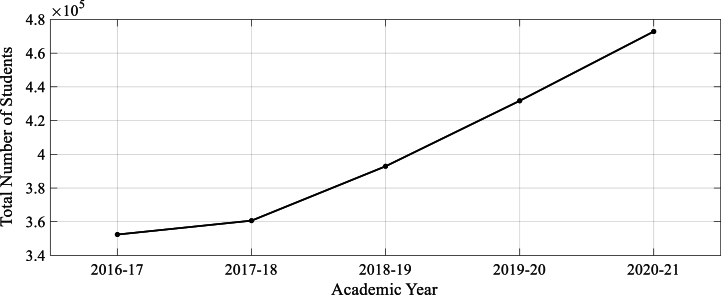
Overall Student's enrollment in different Academic year.

The graphical results in Fig. 3, showcasing annual trends in student enrollments from 2016 to 17 to 2020-21 provides a relevant context for understanding the potential impact of ChatGPT on educational settings in underdeveloped regions[5]. The steady increase in Bachelor's enrollments, along with the relatively stable numbers for Master's and PhD programs, indicates a growing student population that could benefit from innovative educational technologies like ChatGPT.

**Fig. 4:**
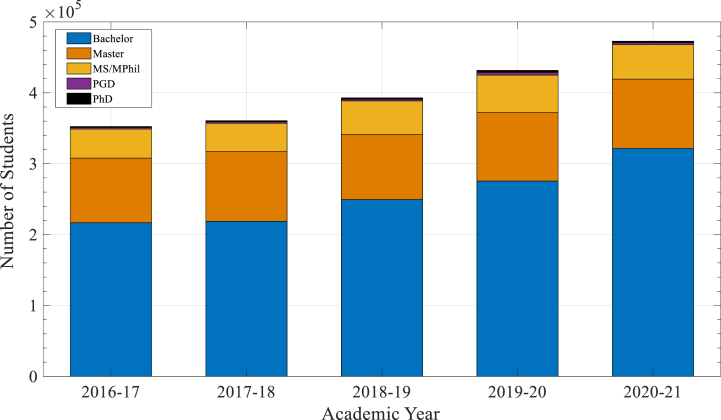
Distribution of students across degree categories.

Fig. 4, illustrates significant growth in Bachelor's enrollments, from 216,657 in 2016-17 to 321,563 in 2020–21. This upward trend highlights the increasing demand for higher education, which ChatGPT can support by offering scalable and personalized learning experiences, especially in regions with limited educational resources. For Master's and PhD programs, where enrollments have shown stability, ChatGPT can assist in specialized areas such as research support, automated feedback, and administrative efficiency, enhancing the quality of advanced education.

**Fig. 5:**
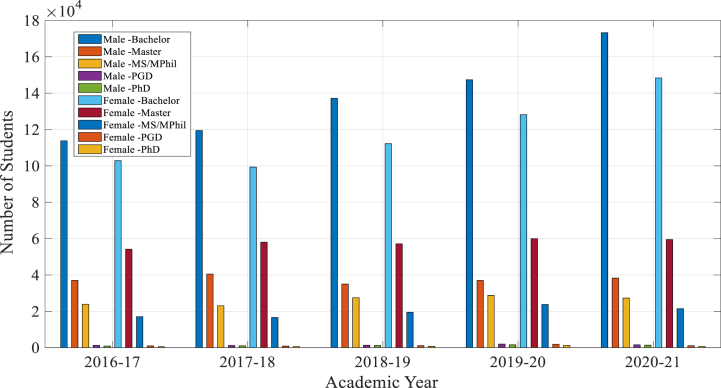
Male and Female students by degree type each year.

Fig. 5, presents the distribution of male and female students across different degree categories, reflecting a gender-balanced growth in student numbers. This balanced distribution underscores the importance of equitable access to educational resources. ChatGPT can play a crucial role in promoting educational equity by providing personalized learning opportunities to both male and female students, ensuring that all students receive the support they need to succeed.

Overall, the data provide a comprehensive overview of how student enrollments in different degree programs have evolved over the past five years in Pakistan. They highlight both overall trends and gender-specific distributions across Bachelor's, Master's, PhD, and total student populations. By integrating ChatGPT into educational settings, we can leverage these trends to address the increasing demand for higher education, promote equitable access to educational resources, and enhance the quality of education through innovative and personalized learning experiences.

**Fig. 6:**
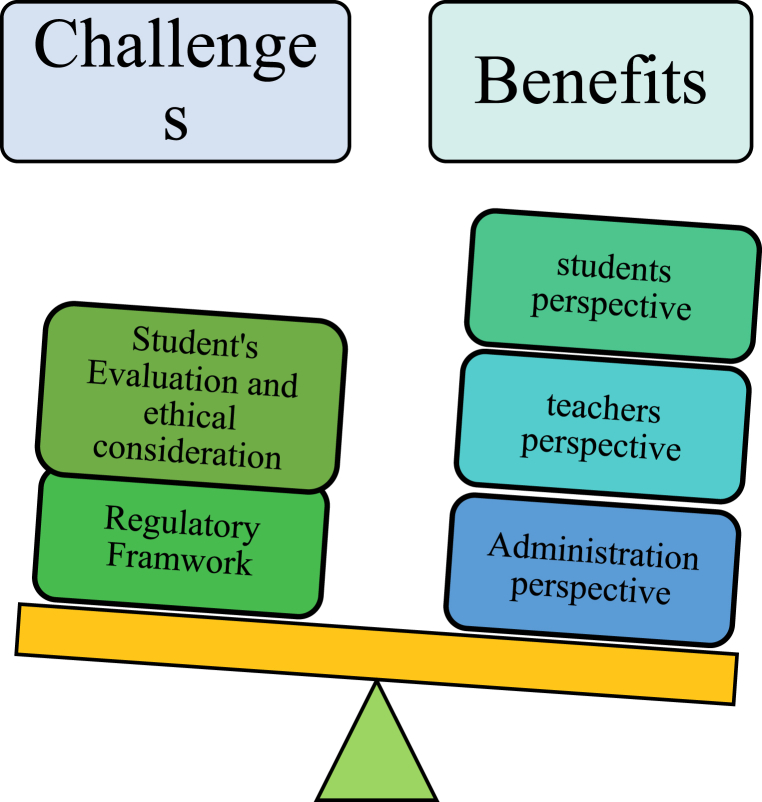
Challenges vs benefits.

Based on the above data, the core challenges of ChatGPT and its benefits are highlighted in Fig. 6. On this basis, the following research questions are formulated as follows.

1. What are the perceived advantages and disadvantages of implementing ChatGPT in educational settings within underdeveloped regions, from the perspectives of teachers, students, and administrators?
2. How can ChatGPT be effectively integrated into the existing educational infrastructure of underdeveloped regions to enhance learning outcomes and educational equity?
3. What are the challenges faced by universities in implementing comprehensive frameworks for integrating advanced technologies, such as ChatGPT, into their educational strategies?

In a nutshell, this research paper aims to shed light on the potential benefits and risks associated with integrating ChatGPT into academic settings in underdeveloped countries. By addressing these questions, we aim to provide insights that can inform educators and policymakers in developing regulatory measures to ensure responsible and ethical use of ChatGPT while fostering academic advancement in these unique contexts.

## 4 Methodology

The methodology for addressing these research questions highlighted in Section 3 involved conducting a systematic literature review. Peer-reviewed articles, conference papers, and relevant reports were extensively searched across academic databases. The review focused on the benefits and challenges of integrating ChatGPT in educational contexts, particularly within underdeveloped regions.

### 4.1 Exploring perceived advantages and disadvantages

ChatGPT isn't just a fancy tech tool; it's your go-to buddy for learning and solving problems. Need help with math? It's got your back. Struggling with coding? ChatGPT can guide you through it. Using ChatGPT as a teaching assistant provides advantages such as round-the-clock support availability, personalized learning tailored to individual needs, and streamlined access to educational resources and feedback. However, challenges include the risk of over-reliance fostering dependency, limitations in delivering nuanced responses to complex queries, concerns regarding information accuracy, and ethical considerations related to biases in its responses.

**Fig. 7:**
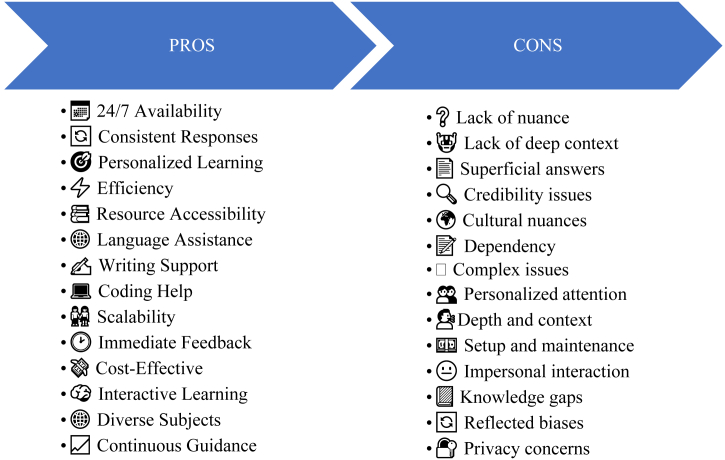
Pros and cons of ChatGPT/AI in academia.

Balancing these benefits and challenges requires meticulous integration and oversight to optimize ChatGPT's educational benefits effectively. Fig. 7, represents the positive and negative aspects of ChatGPT in academia.

In the subsequent sections, we will systematically address each of these concerns, focusing on the integration of ChatGPT from both the students' and teachers' perspectives. We will evaluate ChatGPT's performance in educational settings, analyze its impact on learning outcomes, and propose strategies to maximize its benefits while addressing potential challenges.

#### 4.1.1 From students’ perspective

ChatGPT plays a pivotal role in transforming student productivity and boosting overall academic performance. By offering personalized learning experiences, the model adapts to individual student needs, providing tailored assistance and explanations. This personalized approach enhances comprehension and engagement, ultimately fostering a more conducive learning environment. Additionally, ChatGPT serves as a valuable resource for students seeking instant clarification on complex topics or assistance with assignments. Its ability to generate coherent and contextually relevant responses in natural language facilitates efficient communication, enabling students to overcome learning hurdles promptly. As a result, the integration of ChatGPT in education contributes to a significant enhancement in student productivity and academic achievement, laying the foundation for a more effective and adaptive learning experience.

A) Transforming Student Productivity

Addressing the persistent challenge of low productivity in higher education is of particular significance in the context of third-world countries, where resource constraints often exacerbate these issues. Research studies, including those by Kasneci et al. (2023) and Fauzi et al. (2023), highlight the potential of ChatGPT to significantly enhance students' productivity in such settings. By providing valuable resources, improving language skills, increasing learning efficiency, promoting collaboration, and boosting motivation, ChatGPT emerges as a promising tool to address productivity challenges in the unique educational landscape of third-world countries.

Malik et al. [12] echoes these findings but underscore the importance of responsible use, particularly in regions with limited resources. They advocate for adherence to regulations and guidelines to safeguard academic integrity and prevent overreliance on ChatGPT. In third-world countries, where educational resources may be scarce, responsible utilization of technology becomes crucial for maximizing its benefits.

It is crucial to recognize, as highlighted by Lo [3], that ChatGPT's performance may vary across subject domains. While excelling in economics, it may fall short in mathematics. This nuance is particularly relevant for educators in third-world countries who must tailor the integration of ChatGPT to align with the specific challenges and requirements of their academic programs.

While ChatGPT can undoubtedly assist instructors in generating materials and guiding students, the latter requires training in its responsible use. This training becomes imperative not only to optimize the benefits of the technology but also to address concerns related to plagiarism and the dissemination of misinformation, ensuring the maintenance of academic integrity in the unique context of third-world educational settings [3].

In navigating the integration of ChatGPT in third-world countries, a delicate balance must be struck between leveraging its capabilities to enhance productivity and fostering a responsible and ethical educational environment. By doing so, educators can harness the potential benefits of ChatGPT while addressing the specific challenges posed by low productivity in the context of resource-limited educational settings.

B) Boosting Student Performance

ChatGPT3, with its potential to support students in meeting their basic psychological needs of autonomy, competence, and relatedness, holds promise for enhancing self-determination and academic motivation and performance (Geary et al., 2023; Ryan & Deci, 2000). By offering a sense of control over writing, it addresses autonomy needs, challenging the assumption that it is solely used for writing assignments (Pavlik, 2023; Perkins, 2023). The crucial question arises: can ChatGPT3 support academic writing without compromising the student's voice, distinct from institutional or professional editing support?

The autonomy granted to students by ChatGPT3 is pivotal. This AI model goes beyond mere assistance, potentially fostering a sense of competence by imparting skills and resources for specific writing tasks. When students engage in ChatGPT3 for written assignments, they gain control over their writing process and outcomes. This empowerment enables idea generation, structural planning, and feedback reception, leading to increased autonomy and mastery over writing abilities. It is imperative, however, to recognize that ChatGPT3 should complement, not replace, the development of students' writing skills and critical thinking. Long-term success hinges on nurturing these foundational skills.

The potential for ChatGPT3 to cultivate relatedness among university students is noteworthy. In an era where increasing numbers of students feel lonely and isolated (Thomas et al., 2020), ChatGPT3 could provide a form of social support. With real-time, 24/7 availability for feedback and suggestions, it can address a void in student support, offering assistance when human interaction is unavailable. It is crucial to emphasize that ChatGPT3 should not replace human connections but rather supplement them during times of unavailability.

Addressing productivity concerns in higher education, ChatGPT3 emerges as a tool that can enhance student efficiency by providing resources, improving language skills, and fostering collaboration (Kasneci et al., 2023; Fauzi et al., 2023). However, responsible use is paramount to combat overreliance. Studies, such as those by Malik et al. [12], advocate adherence to regulations and guidelines to uphold academic integrity. Table 2. Summarizes ChatGPT Potential in Supporting Students in Academia.

**Table 2:** Summary of ChatGPT potential in supporting students.

References	Aspect	Key Points
Ryan & Deci, 2000	Overall, Purpose of ChatGPT3	• Enhances self-determination • Boosts academic motivation and performance • Supports autonomy, competence, and relatedness
Pavlik, 2023; Perkins, 2023	Independence of decision-making	• Provides control overwriting • Challenges used for only writing assignments Grants pivotal autonomy for students
		• Fosters a sense of competence • Imparts skills and resources for writing tasks Enables autonomy in idea generation, planning, and feedback
		• Fosters a sense of competence • Imparts skills and resources for writing tasks Enables autonomy in idea generation, planning, and feedback
Thomas et al., 2020	Relatedness Among Students	• Cultivates relatedness for lonely, isolated students • Provides 24/7 feedback availability, addressing support void
		• Supplement connections during the unavailability of physical resources
Kasneci et al., 2023; Fauzi et al., 2023 [12];	Academic Productivity	• Addresses productivity concerns in higher education • Enhances efficiency through resources and language skills improvement • Fosters collaboration
		• Paramount responsible use to combat overreliance • Advocates adherence to regulations and guidelines for academic integrity
		• Subject domain variations exist.

### 4.2 From Teacher's perspective

In the world of education, ChatGPT is like a high-tech sidekick, helping students in less wealthy countries with writing, language learning, and personalized lessons. It's like a friendly guide that adapts to each student's needs.

But, there's a challenge – some students cheat, just like in the old days when someone brought notes into an exam. Nowadays, with online tests, it's tricky to make sure students are really doing the work. Some people suggest punishing cheaters, but that doesn't always solve the problem.
Now, here's where teachers come in. Teachers are magicians, good teachers can guide students on how to use ChatGPT in the right way. They help students think critically, work together, and learn effectively. When asked if good teachers can make students use ChatGPT better, the answer is yes! Teachers play a big role in making sure students use technology wisely.
But, there's a bigger picture. To stop cheating and make sure students do the right thing with ChatGPT, it's not just about punishment. It's about building good character in students. Imagine teachers showing students how to be honest, make good choices, and think for themselves. That's the key to a brighter future.
So, in this journey of learning and technology, teachers are the heroes' guiding students. They make sure ChatGPT is a helpful friend, not a sneaky shortcut. By building good character, teachers create a strong foundation for students to succeed – not just in exams, but in life.

Numerous potential applications of ChatGPT in classrooms have been identified. A study by Zhai [13] outlines three primary ways in which ChatGPT can be integrated into education: personalizing learning, automating administrative tasks, and assisting in teaching. According to an employee at ChatGPT, the technology is already employed in classrooms for lesson development, designing quiz questions, and providing feedback on students' writing (Stanford, 2023). Aoun (2017) introduces the concept of humans, suggesting that technology, including ChatGPT, can assist students in developing strengths for a future labor market where they will collaborate with capable technologies (INSEAD, 2023).

Terwiesch [14] evaluates ChatGPT's performance in MBA assessments, revealing that ChatGPT performs remarkably well in students' examinations, achieving average grades of B for all questions. The study highlights the potential applications of ChatGPT in education, such as quickly generating exam questions, albeit with the need for human intervention. Another study using ChatGPT for microeconomics examination questions found mixed results, with the technology answering only one question correctly out of twenty (INSEAD, 2023). The study emphasizes the importance of students critically evaluating ChatGPT's responses and not solely relying on them.

Furthermore, ChatGPT has been tested in a law school setting, demonstrating proficiency in writing essays but lacking the ability to identify relevant issues or provide sufficient depth compared to student work [15]. Despite its limitations, ChatGPT is viewed as potentially reshaping traditional assignments and assessments in education (Stokel Walker, 2022). In a seminar discussion on ChatGPT's use in the classroom, the technology was recognized as presenting numerous opportunities for education and prompted fundamental questions about the role of educators (Stanford, 2023).Table 3, highlights the impacts of integrating ChatGPT in the classroom from students' perspective.

**Table 3:** Teacher's perspective: Integrating ChatGPT in the classroom.

Reference	Key Integration Areas	Key Performance and Insights
Aoun (2017)	Humanics Concept, Future Labor Market Skills	ChatGPT aids students in developing skills for future collaboration.
StokelWalker (2022)	Reshaping Assignments and Assessments	Viewed as transformative despite limitations; prompts rethinking of education.
Zhai [13]	Personalizing Learning, Automation, Teaching	Identifies primary integration areas for ChatGPT in education.
Terwiesch [14]	MBA Assessment Performance	Performs well, achieving average grades of B in student examinations.
INSEAD (2023)	Exam Question Generation, Microeconomics	Mixed results; underscore the need for human intervention and evaluation.
Choi [15]	Law School Essays	Proficient in writing but lacks depth in identifying relevant issues.
Stanford (2023)	Lesson Development, Quiz Design, Feedback	Technology is employed for diverse educational tasks in classrooms.
Stanford (2023)	Seminar Discussion	Recognized for presenting numerous opportunities in education.

#### 4.2.1 A teaching assistant

According to Alshater [16], ChatGPT isn't limited to academics. It can give you advice on health, time management, and more. Plus, it's super handy for coding tasks, helping you fix those tricky Python errors (INSEAD, 2023). In today's world, everyone needs to understand tech. ChatGPT isn't just for school; it's your ticket to better products and services in all sorts of jobs. It helps with marketing, operations, legal questions, and even healthcare discoveries [17]. Aoun (2017) says the future needs people who understand tech and people. ChatGPT is here to help you build those skills. It's not just a study tool; it's your partner in driving innovation. So, whether you're studying, coding, or exploring new ideas, ChatGPT is the friend that makes learning easy and fun. Table 4, summarize the positive and negative impacts of using ChatGPT as a teaching assistant.

**Table 4:** Pros and Cons of using ChatGPT as a teaching assistant.

Reference	Pros	Cons (Overall content)
Aoun, 2017	Emphasizes the importance of understanding both tech and people for the future. ChatGPT aids in building essential skills for driving innovation.	• Potential for cheating in online tests due to ChatGPT assistance. • Teachers face the challenge of guiding students on the appropriate use of ChatGPT and ensuring responsible technology use. • Need to move beyond punishment and focus on character building to ensure responsible technology use. • The portrayal of ChatGPT as a helpful friend may lead to overreliance, potentially hindering independent thinking and creativity. • The challenge of ensuring ChatGPT is used as a tool for learning and problem-solving, not as a shortcut or substitute for critical thinking. • Integration of ChatGPT as a teaching assistant requires clear guidance from teachers to maximize its benefits effectively.
[17]	ChatGPT is not limited to academics; it is applicable in various professional domains, including marketing, operations, and healthcare discoveries.	
INSEAD, 2023	ChatGPT is valuable for coding tasks, assisting in solving tricky Python errors.	
[16]	ChatGPT serves beyond academics, offering advice on health, time management, and more. Particularly useful for coding tasks.	

## 5 Potential impact of AI tools on education

In addition to advancing technical knowledge, AI tools like ChatGPT hold significant promise in less economically privileged regions. These technologies have the potential to assist with creative tasks, generating various forms of content such as text, videos, audio, images, and simulations. This could bring about a positive shift in how people in third-world countries create and consume information [18].

In areas where resources may be limited, the ability to produce digital content has become increasingly crucial for both businesses and students. However, not everyone possesses the skills to craft compelling content. AI offers a solution by helping individuals in these regions transform their creative ideas into tangible outputs that others can understand and appreciate. In the context of education, AI can play a role in helping students generate content based on their unique perspectives. This is particularly valuable for non-native speakers, aiding them in tasks such as editing or translation (INSEAD, 2023).

Moreover, AI can foster increased collaboration among students, creating a more engaging learning environment. Yet, it is essential to approach the use of AI in these regions with a focus on ethics. AI's ability to generate realistic content without clear disclosure could lead to misconceptions and challenges in interpreting digital information. Therefore, it becomes crucial to view AI as a supportive tool that can save time and effort, enabling a broader audience in third-world countries to actively participate in creative processes. For instance, it can assist students in designing products and optimizing business practices, offering potential benefits for economic development [18]. Table 5, provides a concise summary of the key points regarding the potential impact of AI tools, specifically ChatGPT in less economically privileged regions, with corresponding references.

**Table 5:** The potential impact of AI tool.

References	Key Points
[18]	AI tools like ChatGPT hold promise in less economically privileged regions. Potential assistance in creative tasks, generating text, videos, audio, images, and simulations.
	Positive impact on how people in third world countries create and consume information.
INSEAD, 2023	In areas with limited resources, AI aids in producing digital content crucial for businesses and students.
	AI helps individuals transform creative ideas into tangible outputs, particularly valuable for non-native speakers in education.
	Contribution to collaborative learning environments, facilitating increased collaboration among students.
	Emphasis on approaching AI use in these regions with a focus on ethics due to potential challenges in interpreting digital information.
	AI is viewed as a supportive tool, saving time and effort, and enabling broader participation in creative processes.
	Potential benefits for economic development, assisting students in designing products and optimizing business practices.

AI tools, such as ChatGPT, hold significant promise in transforming education globally. They offer opportunities to enhance learning experiences through personalized tutoring, immediate feedback, and access to vast educational resources. In less economically privileged regions, AI can bridge gaps by providing essential educational support, facilitating the creation of digital content, and fostering collaborative learning environments. However, careful consideration is needed to address challenges such as ethical implications, digital literacy, and ensuring equitable access to technology. As AI continues to evolve, integrating these tools responsibly into educational practices can unlock their full potential to empower students, educators, and communities worldwide.

## 6 .university strategies for comprehensive framework

The responsible integration of AI tools like ChatGPT in universities requires a holistic approach that upholds ethical principles, tackles educational challenges, and fosters innovation. AI technologies in education have evolved significantly since the 1970s, spanning from personalized learning tools to administrative systems in schools (Al Braiki et al., 2020; Schiff, 2022; UNESCO, 2021a). However, this advancement has brought forth concerns within educational contexts, such as changes in assessment and curriculum design, disparities in access, and the evolving roles of educators (Pelletier et al., 2022; Popenici & Kerr, 2017; Swiecki et al., 2022; TEQSA, 2023; UNESCO, 2021a). Policies addressing these issues emphasize digital literacy, preservation of traditional teaching values, inclusivity, and enhancing teacher professional development (Southgate, 2020; Luan et al., 2020; Tanveer et al., 2020; Ocaña-Fernández et al., 2019; Wang et al., 2021; UNESCO, 2021b). Despite widespread acknowledgment of these concerns, AI policies in education often lack specific and actionable implementation strategies, primarily focusing on workforce development and training of AI specialists (Schiff, 2022; Gellai, 2022; Feldstein, 2019). This gap underscores the need for comprehensive policy frameworks that effectively address ethical considerations and governance in AI education (Sam & Olbrich, 2023; Schiff, 2022).Table 6, summarize the overview of educational policies used to implement AI technologies in academia.

**Table 6:** Overview of AI technologies and educational policies.

Paper	Key Points
**Al Braiki et al., 2020**	• AI technologies in education have evolved from personalized learning tools to administrative systems in schools.
**Schiff, 2022**	• Concerns in educational contexts include changes in assessment and curriculum design, disparities in access to technology, and evolving roles of educators.
**UNESCO, 2021a**	• Policies emphasize the importance of digital literacy, preservation of traditional teaching values, inclusivity, and teacher professional development.
**Pelletier et al., 2022**	• AI policies often lack specific strategies and primarily focus on workforce development and training of AI specialists.
**Southgate, 2020**; **Luan et al., 2020**; **Tanveer et al., 2020**	• Educational policies need comprehensive frameworks addressing ethical considerations and governance in AI education.

### 6.1 International perspective

The integration of AI technologies in education has shown significant potential worldwide, but the benefits and challenges vary widely across different economic contexts. Developed countries have made strides in leveraging AI for educational enhancement, yet underdeveloped countries face unique challenges that necessitate tailored policy frameworks. Issues such as limited technological infrastructure, unequal access to digital resources, and cultural sensitivities require specific attention to ensure AI technologies benefit all segments of society equitably. A comprehensive AI policy framework for underdeveloped countries should prioritize digital inclusion, ethical considerations, capacity building, and adaptation of AI tools to local educational needs. Such frameworks can empower these nations to harness AI's transformative potential in education and bridge the digital divide.

The UNESCO framework for AI in education focuses on putting people first, aiming to protect human rights and equip individuals with essential skills for sustainable development.[6]. It promotes collaboration between humans and AI to enhance learning, work, and daily life. The framework stresses the importance of human control over AI systems to empower teachers and students. Additionally, UNESCO advocates for AI applications that are ethical, transparent, fair, and accountable. Specific recommendations for policymakers are outlined in UNESCO's AI and Education guidance document, reflecting these principles.

For underdeveloped countries, adopting and implementing this framework is particularly critical. It can help address educational challenges, promote inclusive access to technology, and ensure that AI tools meet local needs effectively. By following UNESCO's guidance, policymakers in underdeveloped countries can establish policies that support ethical AI integration and maximize its benefits for education and societal development.

This Table 7, outlines key recommendations derived from UNESCO's framework for integrating AI into education[7]. Each recommendation addresses critical aspects necessary for the effective and ethical adoption of AI technologies in educational settings.

**Table 7:** Summary of UNESCO recommendations for implementing AI in education.

Recommendations	Description
**Interdisciplinary Collaboration**	Collaborate across sectors (education, technology, ethics) to develop comprehensive AI policies that consider all aspects of AI in education.
**Equitable and Inclusive AI Policies**	Develop policies ensuring fair access to AI technologies, addressing biases, and promoting inclusive educational practices.
**Comprehensive AI Master Plan**	Create a detailed plan for AI integration in education, focusing on management, teaching, learning, and assessment with clear objectives and milestones.
**Pilot Testing and Evaluation**	Conduct pilot projects to test AI tools in diverse educational contexts, monitoring their effectiveness and gathering evidence for scaling successful initiatives.
**Support for Local AI Innovations**	Encourage local development of AI solutions tailored to specific educational needs and cultural contexts, supporting startups and researchers in this endeavor.

### 6.2 Proposed framework for university policies on AI tools

**Fig. 8:**
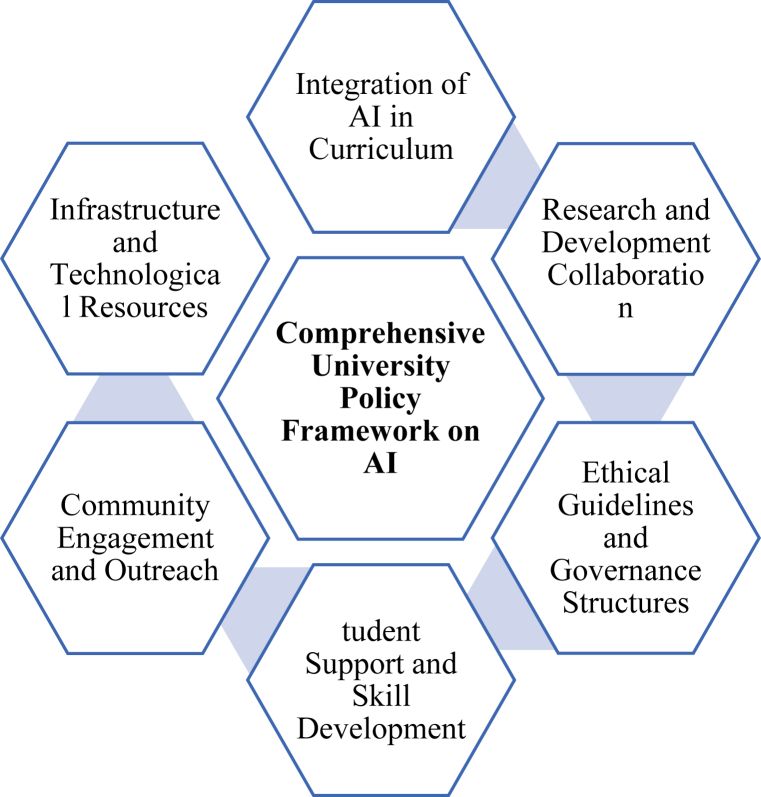
Strategies for university policy framework on AI

Underdeveloped countries face unique challenges such as limited digital infrastructure, insufficient training for educators and students, and concerns about data privacy and ethical use. These challenges necessitate a comprehensive framework that addresses the specific needs of these regions, ensuring that AI tools are accessible, effective, and ethically employed. This proposed framework for university policies on AI tools aims to provide a strategic roadmap for higher education institutions in underdeveloped countries. It focuses on key areas including improving access and infrastructure, providing training and capacity building, establishing ethical guidelines, integrating AI into curricula, supporting research and development, formulating robust policies, promoting sustainability, and ensuring continuous monitoring and evaluation. By implementing this framework, universities can harness the power of AI to enhance educational outcomes, promote equity, and drive innovation. This initiative not only supports the immediate academic community but also contributes to the broader goal of societal development by equipping future generations with the skills and knowledge required to thrive in an increasingly digital world. Fig. 8 represents the key aspects of a comprehensive university policy framework for the implementation and management of AI. Table 8 outlines potential university strategies for a comprehensive framework.

**Table 8:** Summary of University Policy for AI implementation and management.

Strategy	Description
**Integration of AI in Curriculum**	Incorporate AI courses and modules across disciplines to prepare students for AI-driven industries.
**Research and Development Collaboration**	Foster partnerships with industry and research institutions to advance AI technologies and applications.
**Ethical Guidelines and Governance Structures**	Establish policies and guidelines to ensure ethical AI use, addressing issues like bias and privacy.
**Student Support and Skill Development**	Offer training and resources to develop AI skills among students, promoting innovation and readiness for the workforce.
**Community Engagement and Outreach**	Engage with local communities to promote AI literacy and leverage AI for social impact and sustainability.
**Infrastructure and Technological Resources**	Invest in AI infrastructure and resources to support research, teaching, and practical applications.

#### 6.2.1 Integration of AI in curriculum

Universities globally are integrating AI courses across disciplines to equip students with essential skills for AI-driven industries. Align AI integration with university values by promoting ethics, accountability, and transparency [19]. UNESCO emphasizes a human-centric approach, ensuring that AI education prepares students worldwide, including those in underdeveloped countries, for future careers and innovation. This approach aims to bridge educational gaps and empower communities to participate in the global digital economy.

#### 6.2.2 Research and development Collaboration

Collaborations between universities, industry, and research institutions drive AI advancements and their practical applications. UNESCO supports international partnerships to foster knowledge exchange and technology transfer, crucial for underdeveloped countries to access and adapt AI innovations to local contexts. Such collaborations accelerate development goals and empower local researchers and industries. Embrace holistic learning ecosystems, such as Classroom 4.0, to bridge the gap between industry demands and education [20]. Define university strategies that align with the transformative goals of Education 4.0, emphasizing global citizenship, innovation, technology skills, and interpersonal development [21].

#### 6.2.3 Ethical guidelines and governance structures

Establishing ethical guidelines safeguards against misuse of AI technologies within educational settings. UNESCO advocates for inclusive governance that ensures AI benefits are equitably distributed and respects local values. In underdeveloped countries, robust ethical frameworks build trust and acceptance, facilitating responsible AI adoption and innovation aligned with community needs.

#### 6.2.4 Student support and skill development

Universities offer training programs to enhance AI literacy and skills among students, preparing them for AI-related careers and entrepreneurship. UNESCO promotes digital literacy and skill development globally, ensuring underdeveloped countries benefit from AI education to drive economic growth and societal advancement.

#### 6.2.5 Community engagement and Outreach

Engaging local communities in AI initiatives fosters understanding and collaboration. UNESCO encourages universities to demonstrate AI's societal benefits, promoting inclusive AI adoption that addresses local challenges. Community involvement enhances AI relevance and accessibility, empowering communities to leverage technology for sustainable development.

#### 6.2.6 Infrastructure and technological resources

Investing in AI infrastructure supports research, teaching, and practical applications within universities. UNESCO advocates for equitable access to AI technologies, particularly in underdeveloped regions, to enhance educational outcomes and competitiveness. Improved infrastructure bridges digital divides, enabling universities to harness AI for local development and global engagement.

Therefore, adopting UNESCO's guidelines enables universities, especially in underdeveloped countries, to effectively integrate AI into education. By focusing on ethical governance, collaborative research, skill development, community engagement, and infrastructure enhancement, educational institutions can leverage AI to advance learning outcomes, drive innovation, and promote sustainable development globally. In conclusion, the strategic integration of AI in universities involves balancing innovation with ethical considerations and fostering an environment that prepares students for the future. By aligning strategies summarized in Table 8, with ethical values, promoting personalized learning, and staying attuned to evolving regulatory landscapes, universities can leverage AI to enhance education while maintaining a commitment to core educational principles.

## 7 Conclusion

In conclusion, the integration of ChatGPT3 and similar AI technologies in education presents a dynamic landscape of opportunities and challenges. The paper has explored various dimensions, ranging from the benefits and challenges in higher education globally to the specific implications in underdeveloped countries, with a focus on the Pakistani academic context. The literature review emphasized the importance of responsible use, character development, and the potential impact on cognitive development in regions with limited educational resources.

The study highlighted the multifaceted applications of ChatGPT in classrooms, spanning from personalized learning and administrative tasks to content development and addressing productivity challenges. It acknowledged the crucial role of teachers in guiding students to use ChatGPT responsibly and fostering good character to prevent misuse. Furthermore, the research questions identified key areas for exploration, emphasizing the need for understanding the advantages, challenges, and regulatory measures in underdeveloped countries, particularly Pakistan.

The benefits of ChatGPT were discussed in the context of transforming student productivity, boosting academic performance, and acting as a teaching assistant. Additionally, a potential regulatory framework has been proposed to guide the ethical adoption of AI technologies in educational settings, ensuring alignment with educational values and promoting equitable access to technological advancements.

## 8 .future recommendations

To bring ChatGPT and AI into universities, it's crucial to do it right. First, universities need clear rules and values for using AI, ensuring it aligns with their principles. Staying updated on rules and guidelines is vital to make sure everything stays responsible. Next, universities should focus on teaching skills that AI can't do, like thinking critically and being creative. Adopting new ways of learning, like Classroom 4.0, helps students be ready for the future job market. When using AI, make sure it helps students individually and doesn't replace the need for human interaction and teamwork. Personalized learning, tailored to each student, is key to a better learning experience. Lastly, while using technology to engage students, it's important to consider their well-being and the long-term impact. Universities should also invest in researching how AI affects education, ensuring it's used in the best way possible. In summary, the strategic integration of ChatGPT and AI technologies in universities requires a balanced approach that prioritizes ethical considerations, promotes personalized learning, and prepares students for the future. By aligning strategies with ethical values, universities can harness the potential of AI to enhance education while maintaining a commitment to core educational principles.

## Ethics Approval

This article does not contain any studies involving animals performed by any of the authors and human participants performed by any of the authors.

## Data availability Statement

All data supporting the findings of this study are contained within the manuscript.

## Authorship contributions

Amna Shabbir played a key role in conceptualizing, investigating, developing the methodology, validating findings, and drafting the manuscript. Safdar Rizvi provided valuable contributions through reviewing and editing the manuscript. The research was supervised by Mansoor Alam. Mazliham Mohd Su’ud secured funding for the research.

## Declaration of competing interest

The authors declare that they have no known competing financial interests or personal relationships that could have appeared to influence the work reported in this paper.
